# Laparoscopic Banded Gastric Bypass: A Case Report of a Successful Weight Loss Despite Gastro-Gastric Fistula

**DOI:** 10.7759/cureus.42967

**Published:** 2023-08-04

**Authors:** Payam Maaroof, Aslanbi Tezekbay, Lokeshwar Raaju Addi Palle, Jane Kim, Jeffrey J Kraft

**Affiliations:** 1 General Surgery, Hackensack Meridian Health - Palisades Medical Center, North Bergen, USA; 2 Surgery, Kamala Children's Hospital, Chennai, IND

**Keywords:** laparoscopic adjustable gastric band, open roux-en-y gastric bypass surgery, roux-en y, laparoscopic gastric bypass, gastrogastric fistula, banded gastric bypass

## Abstract

Obesity is a global health issue, Roux-en-Y gastric bypass (RYGB), is an effective treatment for weight loss. However, some patients experience insufficient weight loss after RYGB, leading to alternative strategies such as adding an adjustable gastric band to the bypass. This case reports a 43-year-old female with morbid obesity who underwent open RYGB in 2004, achieving significant weight loss. However, she experienced weight regain, indicating RYGB failure. A laparoscopic band was placed around her bypass with no post-operative complications and successful steady weight reduction. During follow-up, an upper gastrointestinal series revealed a gastro-gastric fistula. Despite the fistula, the patient maintained a steady weight, with a significant excess weight loss of 40.2% since the banded gastric bypass surgery. The development of a gastro-gastric fistula, which typically affects weight loss outcomes, was managed conservatively without impacting the patient's steady weight maintenance. This highlights an unexpected weight loss outcome in a patient who underwent laparoscopic banding following RYGB failure and later developed a gastro-gastric fistula. Despite the initial RYGB failure, the patient achieved significant weight loss, surpassing the average reported in previous studies.

## Introduction

Obesity is a significant global health issue, and bariatric surgery remains the sole reliable treatment option for achieving sustainable and appropriate weight loss [1]. Roux-en-Y gastric bypass (RYGB) is widely regarded as one of the most frequently performed bariatric procedures worldwide [2]. It is highly effective in treating obesity, leading to long-lasting weight loss and improved comorbidities such as hypertension, dyslipidemia, type 2 diabetes mellitus, metabolic syndrome, sleep apnea, deep vein thrombosis, and cancer [3].

However, despite that, reports indicate a failure rate of up to 41% for RYGB [2]. Some patients may experience insufficient weight loss due to several factors, such as post-operative lifestyle choices, metabolic dysfunction, mental disorders, or, in rare cases, anatomical issues like long-term outcomes, including dilation of the gastric pouch, gastroenterostomy, or the development of a gastro-gastric fistula between the pouch and the gastric remnant [4]. To address these outcomes, different strategies have been developed, ranging from conservative approaches to surgical interventions. One approach involves altering the size of the pouch and outlet through the addition of a laparoscopic adjustable gastric band also known as banded RYGB [4]. Outcomes of banded RYGB after a failed primary RYGB have reported varying results of excess weight loss (EWL) with a median of 37.6% in long-term follow-ups [3]. Among the studied complications of banded RYGB after a failed RYGB includes late complication of band dysfunctions, port dysfunctions, small bowel obstruction, band-related obstruction, intolerable dysphagia, incisional hernia as well as gastro-gastric fistula, which can create a passage for food to the main stomach portion that results according to current opinion, in a poor weight loss [5-8]. The aim of this case is to present an unexpected weight loss outcome observed in a patient who experienced gastric bypass failure and the development of a gastro-gastric fistula.

## Case presentation

**Figure 1:**
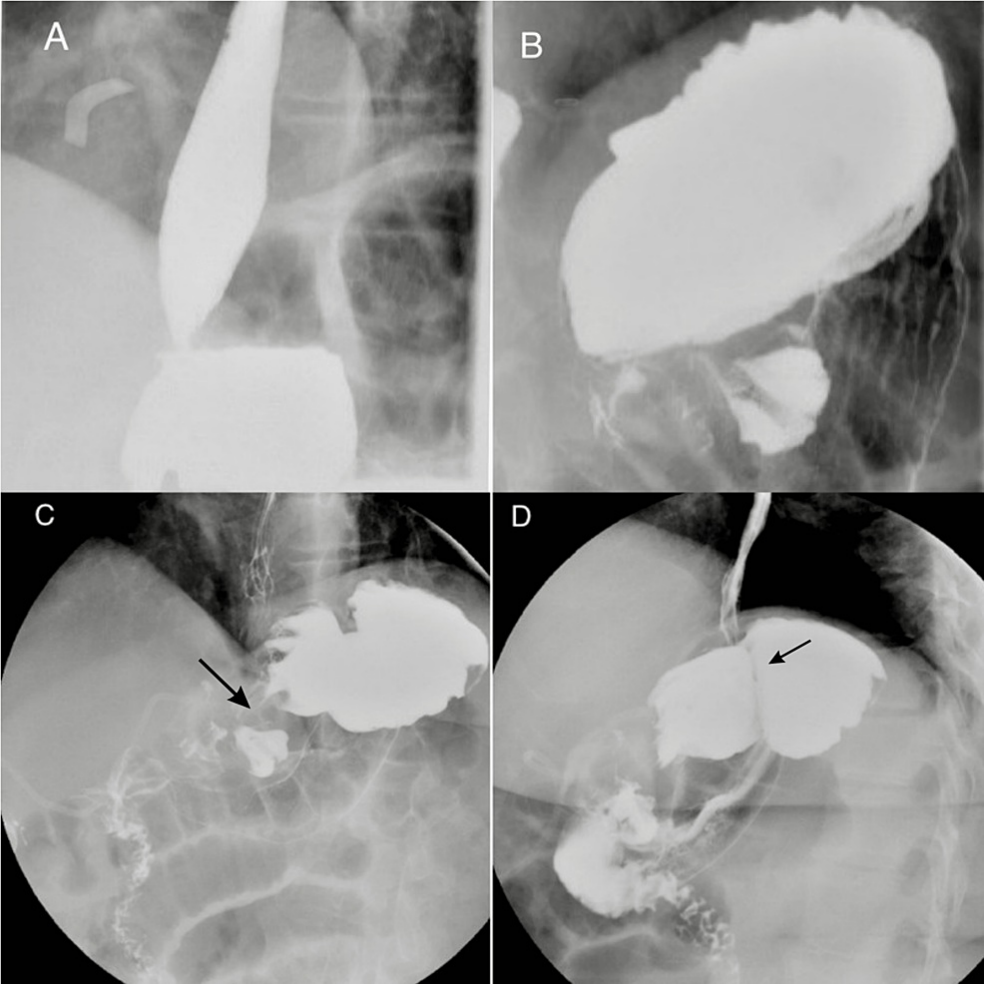
Double contrast upper gastrointestinal series image (A) Esophagogastric junction, (B) distended gastric pouch, (C) A communication/fistula (black arrow), and (D) gastric lap band in the left upper quadrant (black arrow)

A 43-year-old female with a past medical history of morbid obesity, hypertension, and anemia presented with an initial weight of 318 lb and a height of 5 ft 6 in, resulting in a body mass index (BMI) of 51.3 at the time of her open RYGB surgery in 2004. Following the surgery, she achieved a significant weight loss, with a total reduction of 116 lb. As a result, her BMI decreased to 32.6, and she attained 61% of her EWL. During her first appointment at our medical center on February 15, 2021, the patient presented with concerns about weight regain. Her weight had increased to 302 lb, corresponding to a BMI of 48.74, which accounted for approximately 49.5% of her lowest post-operative weight. These findings suggest the failure of her open RYGB surgery. On June 22, 2021, a laparoscopic band was placed around her gastric bypass with no complications following the procedure. Six months later, the patient presented with symptoms suggestive of cholecystitis, and on December 15, 2022, laparoscopic cholecystectomy was performed successfully with no intra-operative or post-operative complications. On April 12, 2023, the patient presented with symptoms of epigastric discomfort after meals. An upper gastrointestinal series was conducted, revealing the presence of a gastro-gastric fistula (Figures 1A-1D). During the patient's most recent follow-up on June 16, 2023, she weighed 241 lb, with a BMI of 38.9, representing a total of 40.2% EWL since her banded gastric bypass surgery.

## Discussion

With the global rise of obesity, bariatric surgery has gained significant popularity over the past few decades as a primary treatment for morbid obesity. Among the various procedures performed in this field, gastric bypass stands out as one of the most widely recognized and established methods [5]. When assessing the progress of a patient's weight loss journey following RYGB surgery, the determination of a successful procedure often relies on calculating EWL. Insufficient weight loss is defined as an EWL of less than 50%, or even weight regain, which is defined as a gain of at least 15% from the lowest post-operative weight [4].

This report presents a case of weight loss observed over a two-year follow-up period following a band procedure after a failed RYGB. Limited studies have investigated the outcomes of weight loss after banded gastric bypass surgery. These studies typically have short follow-up periods of up to three years, and the reported mean EWL is between 28.2% and 37.6% after the second procedure. This places banded bypass at a lower success rate compared to primary procedures such as RYGB or sleeve gastrectomy. Notably, a study by Sohail et al. [2] examined gastric banding as a primary procedure, further supporting this notion.

Based on a similar study conducted by Lazardis et al., the maximum EWL was reported to be at 37.6% after 33.5 months, which is the longest overall follow-up [4]. However, in this case report, the patient achieved an EWL of 40.2%, which represents a higher weight loss within a similar timeframe.

The main concern of our patient (summarized in Tables 1, 2) was initially losing 116 lb, going from 318 lb with a BMI of 51.3 to 202 lb with a BMI of 32.6. And later experienced a weight regain of 100 lb, resulting in a measured weight of 302 lb at that time. This weight regain represents 52.4% of her EWL, categorizing her case as an instance of insufficient weight loss, which can be determined as a failed RYGB procedure [4].

**Table 1:** Surgical history of the patient RYGB - Roux-en-Y gastric bypass

Surgical history timeline	
Open RYGB surgery	2004
Laparoscopic banded bypass surgery	2021
Laparoscopic cholecystectomy	2022
Diagnosis of gastro-gastric fistula	2023

**Table 2:** Summary of patient's weight loss

Weight loss timeline	
Initial weight (2004)	318 lb
Lowest post-operative weight	202 lb
Patient weight when presented to our center (2021)	302 lb
Post-banded bypass weight (current)	241 lb

Considering the weight regain, our patient emerges as a promising candidate for further treatment options as laparoscopic banding of the bypass. As a result, despite the failure of the initial RYGB procedure, the outcome following the banded RYGB resulted in a significant degree of weight loss surpassing the average recorded in previous studies.

Based on patient's follow-ups and during 2022, when the patient was admitted for a laparoscopic cholecystectomy, her preoperative workups indicated no other complications related to the banded bypass. And the patient has maintained a steady weight with no signs of weight regain.

In 2023, the patient presented with mild symptoms suggesting a gastro-gastic fistula, and her diagnosis was confirmed with an upper GI series. Her fistula was treated conservatively since the patient did not show any signs of weight gain and continued to maintain a steady weight during her follow-ups. The latest weight recorded was 241 lb (Table 2).

In contrast to our case, most studies, such as the literature review done by Carrodeguas et al. [6], indicate that complications like gastro-gastric fistula after gastric bypass, which occur in 1%-6% of cases, usually affect the patient's weight loss outcomes. However, cases like ours, in which the symptoms are mild or asymptomatic, are generally managed conservatively. The treatment approach for more severe cases is still a subject of debate [1].

There have been studies focusing on understanding the connection and reasons behind the hormonal changes that occur after the formation of a gastro-gastric fistula compared to repairing the fistula. Based on a case series conducted by O'Brien et al. in 2013, exploring the effect of hormonal changes that occur after primary RYGB and their relation to gastro-gastric fistula, and the changes that occur after the fistula repair showed that fasting and postprandial ghrelin levels decreased and were strongly correlated with weight loss. The insulin response to glucose also tended to increase after GGF repair, but no concomitant increase in GLP-1 was observed. These hormonal changes were observed in a case series, and further studies in this area could help clarify the main mechanisms behind their weight loss effect [9].

## Conclusions

This case report documents an unexpected weight loss result in a 43-year-old female with morbid obesity who had a laparoscopic banded gastric bypass following RYGB failure. Despite initial weight regain, the patient achieved significant weight loss, surpassing the average reported in previous studies. The post-operative development of a gastro-gastric fistula is a complication associated with poor weight loss; however, in this case, it was managed conservatively without impacting the patient's steady weight loss. These findings highlight the efficacy of banded gastric bypass surgery as a treatment option for patients experiencing insufficient weight loss after RYGB. Further research with longer follow-up periods are needed to explore long-term outcomes and optimal management approaches for banded gastric bypass surgery and associated complications such as gastro-gastric fistula.
